# Supplementary material for: Investigation of carbapenemase-encoding genes in Burkholderia cepacia and Aeromonas sobria isolates from nosocomial infections in Iraqi patients
Source: PLoS One. 2025 Aug 29;20(8):e0315490. doi: 10.1371/journal.pone.0315490 (PMC12396648; doi:10.1371/journal.pone.0315490)
Supplement: Attachment — Submitted filename: review report.docx [file pone.0315490.s001.docx]

This study investigates the prevalence of antibiotic resistance, including carbapenem resistance, in *Burkholderia cepacia* and *Aeromonas sobria* isolates from clinical specimens in Iraq. It reported significant resistance rates to multiple antibiotics, with a predominance of blaKPC and blaGES carbapenemase producers, highlighting the need for rapid and accurate bacterial identification methods and better treatment strategies for multi-drug resistant infections.

The manuscript can be accepted only after incorporating following points:

**Major comments**

1. Please revise the title as Investigation of Carbapenemase-Encoding Genes in *Burkholderia cepacia* and *Aeromonas sobria* Isolates from Nosocomial Infections in Iraqi Patients
2. In abstract,

-The **methods** section is vague. Important details such as patient population, or sampling method are missing.

-There is some inconsistency in reporting numbers and percentages. For example You say “75 isolates of *A. sobria* and *B. cepacia* were assessed. *A. sobria* made up 16.6% (n=20), B. cepacia 45.8% (n=55)” — this totals 100% (75 isolates), but the calculation is different 20/75 = 26.6%, 55/75 = 73.3%. There is also a line that mentions “55/57 *recA* gene positive,” but earlier it says only 55 isolates of *B. cepacia* were included.

3. Introduction

- Several sentences repeat similar points (e.g., *B. cepacia*’s resistance and persistence are mentioned multiple times).

"Burkholderiaceae family (previously known as *Pseudomonas cepacian*)" is misleading. Pseudomonas cepacia was the former name of the species, not the family.

- Reference [2] on *B. cepacia* persistence and transmission seems dated or generalized; newer sources might better reflect current understanding of nosocomial spread.

- Consider briefly stating why *A. sobria* (less frequently reported than *A. hydrophila*) is the focus, especially when *A. hydrophila* is mentioned as more common.

-The study objective appears abruptly at the end. The authors are suggested for the transition into the aim more clearly by summarizing the knowledge gap.

4. Methodology Section:

- Although the study employs both VITEK 2 System and recA gene-based PCR for identification, more details on the specific conditions under which these methods were conducted would improve reproducibility. For example, did the VITEK 2 System settings or parameters vary from standard procedures? Similarly, the exact primers used for PCR should be included, along with details on the PCR conditions and any potential limitations of these methods.
- The description of carbapenemase testing through both phenotypic and genotypic approaches is valuable, but further clarification is needed on how results were interpreted in case of discrepancies between the two methods. This would help readers understand the rationale behind the final conclusions on resistance.

5. Discussion of Results:

- The discussion regarding the resistance profiles, especially with respect to *B. cepacia* and *A. sobria*, could be expanded to address the clinical implications of these findings more comprehensively. For example, how do these resistance patterns correlate with patient outcomes or impact therapeutic decisions? Also, how do the findings compare with other studies in the region or globally in terms of therapeutic options?
- The prevalence of carbapenemase-producing strains is discussed, but the manuscript could benefit from more in-depth analysis on the mechanisms of carbapenem resistance. For instance, are there any particular resistance mechanisms (e.g., porin loss, overproduction of β-lactamases) that could explain the observed high levels of resistance in *B. cepacia* and *A. sobria*? The manuscript suggests that other resistance mechanisms could be at play, but this could be explored in more detail.

6. Statistical Analysis:

While the study includes some breakdowns of resistance patterns (e.g., percentage of resistance), the manuscript lacks a detailed statistical analysis of the data. Including confidence intervals, significance testing (e.g., chi-square tests for categorical variables like gender and resistance patterns), and comparative analysis would improve the robustness of the findings.

**Minor Comments and Typos:**

1. **Typo/Grammar:**
   - "No A. sobria was found in any of the cerebrospinal fluid specimens." → "No *A. sobria* isolates were found in any of the cerebrospinal fluid specimens."
   - "This is consistent with Pineda-Reyes et al. (2024), who observed that A. sobri is linked to a variety of human infections..." → "A. sobria" (should match the species name format consistently).
   - Please be consistent in naming species, especially with the genus and species names like *B. cepacia* and *A. sobria*. Use italics throughout and ensure the first letter of the genus is capitalized.
   - Ensure uniform citation formatting throughout the manuscript. There are a few inconsistencies in the way references are presented (e.g., reference numbering).
2. **Figures/Tables:**
   - The manuscript could benefit from including figures or tables that summarize the major findings, such as the resistance rates for each antibiotic tested, the types of specimens sampled, and a comparison of resistance patterns between *B. cepacia* and *A. sobria*. This would make the data more accessible and visually impactful.
3. **Conclusion:**
   - The conclusion is well-written but could be more focused on specific recommendations for future research and clinical practice. A few key research questions or clinical practice guidelines based on the findings would strengthen this section. For example, should clinicians be more cautious when using specific antibiotics for *B. cepacia* and *A. sobria* infections based on the resistance patterns observed?
   - There seems to be some inconsistency in the way data is reported. For example, you mention percentages in some cases but then refer to absolute numbers in others (e.g., "58.7% male" vs. "44 males"). It would be clearer and more standardized to report either both absolute numbers and percentages, or just one for consistency.
