# Supplementary material for: Investigation of carbapenemase-encoding genes in Burkholderia cepacia and Aeromonas sobria isolates from nosocomial infections in Iraqi patients
Source: PLoS One. 2025 Aug 29;20(8):e0315490. doi: 10.1371/journal.pone.0315490 (PMC12396648; doi:10.1371/journal.pone.0315490)
Supplement: Attachment — Submitted filename: Respose to Reviewers 9-5-2025.docx [file pone.0315490.s003.docx]

**Dear Dr. Dwij Raj Bhatta**

**Academic Editor of PLOS ONE**

Subject : Submission of revised manuscript entitled "An investigation of carbapenemase-encoding genes in *Burkholderia cepacia* and *Aeromonas sobria* nosocomial infections among Iraqi patients". (PONE-D-24-53922)

Thank you for your email of 2 May, 2025 enclosing the editor and reviewers ̓comments. We also greatly appreciate the reviewers for their complimentary comments and suggestions. We have carefully reviewed the comments and have revised the manuscript accordingly. Our point to point responses are given below. (The reviewer’s comments are in italics). Changes to the manuscript are indicated in yellow highlighted sentences/words. We hope that you find our responses satisfactory and that the manuscript is now acceptable for publication. **Anyway, we should be grateful if you let us know about our further changes required.**

Yours sincerely,

**Corresponding author**: Mushtak T. S. Al-Ouqaili

Department of Microbiology, College of Medicine, University of Anbar, Al-Anbar Governorate, Ramadi, Iraq Email: ph.dr.mushtak_72@uoanbar.edu.iq

Tel: +9647830014212

The followings are point-by-point responses :

**Response to Editor**

***We note that you have referenced (Hussein RA, Al-Ouqaili MTS, Majeed YH. Association between alcohol consumption, cigarette smoking, and Helicobacter pylori infection in Iraqi patients submitted to gastrointestinal endoscopy. J Emerg Med Trauma Acute Care [Internet]. 2022 Dec 22 [cited 2022 Dec 23];2022(6):12. Available from:*** [***https://www.qscience.com/content/journals/10.5339/jemtac.2022.aimco.12***](https://www.qscience.com/content/journals/10.5339/jemtac.2022.aimco.12)***) which has currently not yet been accepted for publication. Please remove this from your References and amend this to state in the body of your manuscript: (i.e “Bewick et al. [Unpublished]”) as detailed online in our guide for authors***
[***http://journals.plos.org/plosone/s/submission-guidelines#loc-reference-style***](http://journals.plos.org/plosone/s/submission-guidelines#loc-reference-style)

**Response to Editor:**

Thank you for highlighting this important point. We have carefully reviewed and revised the manuscript to ensure uniform citation formatting throughout. All reference numbers have been checked for accuracy and consistency, and the citation style has been standardized in accordance with the journal’s guidelines.

This reference was removed. “Hussein RA, Al-Ouqaili MTS, Majeed YH. Association between alcohol consumption, cigarette smoking, and Helicobacter pylori infection in Iraqi patients submitted to gastrointestinal endoscopy. J Emerg Med Trauma Acute Care [Internet]. 2022 Dec 22 [cited 2022 Dec 23];2022(6):12. Available from: <https://www.qscience.com/content/journals/10.5339/jemtac.2022.aimco.12>” *Responses to Reviewer's Comments:*

***Major comments***

1. ***Please revise the title as Investigation of Carbapenemase-Encoding Genes in Burkholderia cepacia and Aeromonas sobria Isolates from Nosocomial Infections in Iraqi Patients***

***Response to Reviewer:***

I appreciate your insightful comments. Following your suggestion, we have revised the title to " Investigation of Carbapenemase-Encoding Genes in *Burkholderia cepacia* and *Aeromonas sobria* Isolates from Nosocomial Infections in Iraqi Patients." This updated title better reflects the content and focus of our study. Thank you for your recommendations on making the manuscript more accessible.

2.	***In abstract,***

***-The methods section is vague. Important details such as patient population, or sampling method are missing.***

***Response to Reviewer:***

Thank you for your valuable feedback. In response to your comments regarding the clarity of the Methods section, we have revised it to include additional details about specimen sources, identification procedures, antibiotic susceptibility testing, and molecular analyses. We appreciate your helpful suggestions and thank you for your contribution to improving the manuscript.

"A total of 120 clinical specimens from patients with nosocomial infections were collected randomly. Ear swabs, urine, burns, wounds, and cerebrospinal fluid samples were all cultured on selective media. Using biochemical assays, *Burkholderia cepacia* and *Aeromonas sobria* were identified, and the VITEK-2 system confirmed their identities. VITEK-2 was utilized to perform antibiotic susceptibility testing in accordance with CLSI standards. The meropenem-EDTA combination disc test was used to phenotypically screen for the production of metallo-β-lactamase (MBL). The *recA* gene in *B. cepacia* and the genes encoding carbapenemase in both species were found using PCR tests. "

***There is some inconsistency in reporting numbers and percentages. For example You say “75 isolates of A. sobria and B. cepacia were assessed. A. sobria made up 16.6% (n=20), B. cepacia 45.8% (n=55)” — this totals 100% (75 isolates), but the calculation is different 20/75 = 26.6%, 55/75 = 73.3%.***

***There is also a line that mentions “55/57 recA gene positive,” but earlier it says only 55 isolates of B. cepacia were included.***

**Response to Reviewer:**

Thank you for pointing out the inconsistency in the reporting of percentages. We have corrected the statement to reflect the accurate proportions based on the total of 75 isolates, with *A. sobria* comprising 26.6% (n=20) and *B. cepacia* comprising 73.3% (n=55).

**Response to Reviewer**

Thank you for highlighting this difference.. We have clarified that only 55 isolates tested positive for the *recA* gene, although 57 isolates were originally diagnosed as *B. cepacia* using the VITEK 2 technology. The VITEK 2 system may have misidentified the two negative isolates, which can happen with closely similar non-fermenting bacteria.

The text has been updated to reflect that all 55 isolates that the VITEK 2 system identified as *B. cepacia* also tested positive for the *recA* gene. This supports the reliability of the identification and validates their classification within the *B. cepacia* complex. This discovery is now appropriately reflected in the manuscript's updated sentence. We appreciate your careful review and constructive feedback.

1. ***Introduction***

***- Several sentences repeat similar points (e.g., B. cepacia’s resistance and persistence are mentioned multiple times).***

Thank you for your insightful observation regarding the redundancy in the background section. We have revised the text to eliminate any repetitions, particularly concerning the persistence and resistance mechanisms of *Burkholderia cepacia*. While maintaining all the essential background information to provide context for the study, we have streamlined the section for improved clarity and scientific flow. We appreciate your constructive criticism, which has enhanced the overall quality of the manuscript.

“The bacterial isolates of *B. cepacia* frequently show intrinsic resistance to several antibiotics or drugs classes, making infections difficult to treat. This resistance, which includes decreased susceptibility to substances like ticarcillin, cephalosporins, phosphonic acid antibiotics, polymyxins, and aminoglycosides, develops spontaneously and does not require gene acquisition or mutation“

***"Burkholderiaceae family (previously known as Pseudomonas cepacian)" is misleading. Pseudomonas cepacia was the former name of the species, not the family.***

**Response to Reviewer**

Thank you for highlighting the taxonomic inaccuracy. We have revised the text to clarify that Pseudomonas cepacia was the former name of the species, not of the Burkholderiaceae family. The corrected sentence now accurately reflects both the historical classification and the current taxonomy of Burkholderia cepacia. We appreciate your thorough review and valuable feedback.

“Burkholderia cepacia is a Gram-negative, rod-shaped, motile bacterium belonging to the Burkholderiaceae family (formerly known as Pseudomonas cepacia).”

***Reference [2] on B. cepacia persistence and transmission seems dated or generalized; newer sources might better reflect current understanding of nosocomial spread.***

**Response to Reviewer:**

Thank you for your insightful comment regarding the need for a more current reference on Burkholderia cepacia's persistence and transmission. In response, we have updated the manuscript to include a recent and more relevant source: Silva-Santana G, Sales FLS, Aguiar AR, Brandão MLL. Pharmaceutical Contamination by Biofilms Formed of the Burkholderia cepacia Complex: Public Health Risks. Processes. 2025;13(5):1270. <https://doi.org/10.3390/pr13051270>

***Consider briefly stating why A. sobria (less frequently reported than A. hydrophila) is the focus, especially when A. hydrophila is mentioned as more common.***

**Response to Reviewer:**

Thank you for your insightful observation. While A. hydrophila is indeed more frequently reported in clinical settings, our focus on A. sobria arises from its emerging role as a pathogen in nosocomial infections, particularly in the region where our study was conducted. Recent isolates from patients in our cohort have shown a significant presence of *A. sobria*, which warrants further investigation into its resistance patterns and potential public health implications. In the revised manuscript, we have briefly clarified this rationale to provide better context for its inclusion.

“The current study focuses on the prevalence of *A. sobria*, highlighting its increasing clinical significance and justifying its inclusion in this analysis, despite the fact that *A. hydrophila* is more commonly reported in clinical infections.”

***The study objective appears abruptly at the end. The authors are suggested for the transition into the aim more clearly by summarizing the knowledge gap.***

**Response to Reviewer:**

Thank you for your valuable suggestion. We have revised the manuscript to improve the transition into the study's objective by first summarizing the knowledge gap. This enhancement provides clearer context and emphasizes the importance of investigating carbapenemase genes and antibiotic resistance patterns in *B. cepacia* and *A. sobria*. We appreciate your feedback, as it has significantly improved the clarity of the manuscript.

“Although *A. sobria* and *B. cepacia* are becoming more widely recognized as important nosocomial infection pathogens, little is known about the molecular resistance mechanisms of these bacteria, especially in relation to carbapenemase genes. More research on the resistance characteristics of multidrug-resistant bacteria is essential due to their rising prevalence. The aim of this study was to investigate the occurrence of carbapenemase genes and patterns of antibiotic resistance in isolates of *B. cepacia* and *A. sobria*.”

***4- Methodology Section:***

***-	Although the study employs both VITEK 2 System and recA gene-based PCR for identification, more details on the specific conditions under which these methods were conducted would improve reproducibility. For example, did the VITEK 2 System settings or parameters vary from standard procedures? Similarly, the exact primers used for PCR should be included, along with details on the PCR conditions and any potential limitations of these methods.***

**Response to Reviewer:**

We appreciate the reviewer’s valuable feedback regarding the need for increased methodological clarity. In response, we have revised the Methods section to include more detailed information on the identification procedures. Specifically, we now specify that the initial identification was based on traditional bacteriological methods, which included Gram staining, oxidase testing, motility testing, and growth on selective media. Final confirmation was performed using the VITEK® 2 Compact B System (BioMérieux, France) with VITEK® 2 GN ID cards, in accordance with the manufacturer’s guidelines. No modifications or deviations from the standard operating procedure were made. The instrument automatically processed bacterial suspensions prepared according to BioMérieux’s instructions, ensuring consistency and reproducibility across all tested isolates. These enhancements aim to improve the reproducibility and transparency of the identification process.

“Gram-negative isolates suspected of being *A. sobria* or *B. cepacia* were initially identified using normal bacteriological techniques. According to MacFaddin's (2000) procedures, these included Gram staining, oxidase and motility tests, and growth assessment on selective medium such MacConkey agar, blood agar, and mannitol salt agar. Morphological and microscopic features were also assessed. The VITEK® 2 Compact B System (BioMérieux, France) with VITEK® 2 GN ID cards was used to confirm the final species-level identification in accordance with the manufacturer's instructions.”

**Response to Reviewer:**

We appreciate the reviewer's insightful recommendation. In response, we would like to clarify that the manuscript already includes detailed descriptions of the PCR protocols used for detecting both the *recA* gene and the carbapenemase genes. These descriptions specify the primer sequences, product sizes, annealing temperatures, and the exact thermal cycling conditions for each gene target, as well as the reagents and volumes used in the reaction mixtures (see Tables 1 and 2). This level of detail was included to ensure transparency and enhance the reproducibility of our methods. Additionally, we have cited relevant references and indicated the use of validated primers. We are happy to provide further information on these topics if needed.

Additionally, a brief assessment of these strategies' limitations has been added. However, low template concentration, degraded DNA quality, or mutations at primer binding sites can all cause traditional PCR to produce false-negative findings. Furthermore, conventional PCR is unable to distinguish between genes situated on chromosomes and those placed on plasmids, nor does it reveal information on gene expression.

Brisse and colleagues discovered that, despite the fact that B. gladioli is not listed in the VITEK2 database (and so should have a higher probability of misidentification), only one isolate of the bacteria was recognized by the VITEK 2 apparatus as "*B. cepacia* or *B. pseudomalleii*." However, as other isolates of these species or other non-fermenters like Achromobacter or Alcaligenes may be mistakenly recognized as B. cepacia, it is always an excellent choice to use a molecular approach to validate the B. cepacia identification [25]. The recA gene has been widely used in bacterial systematics and has proven to be highly helpful in identifying species of the B. cepacia complex. By using phylogenetic analysis of sequence variance within the gene, it is possible to distinguish between the nine present species of the B. cepacia complex

**PCR techniques for *recA* gene detection in *B. cepacia* isolates**

Using the *recA* gene of the Bcc complex in clinical samples and BCR1 and BCR2 primers, a standard PCR technique was used to directly molecularly identify the Bcc complex in all 75 extracted DNA samples (Table 1). A 25 μl reaction system comprising 12.5 μl of GoTaq® Green Master Mix (2X) (Promega, USA), 1 μl of each forward and reverse primer, 7.5 μl of nuclease-free water, and 3 μl of extracted DNA template was used for the PCR experiments. For the *recA* gene, the PCR reaction protocol was set up as follows: 30 cycles of 45 seconds at 94 ^O^ C, 45 seconds at 56 ^O^ C, and 90 seconds at 72 ^O^ C were followed by one cycle of 94 °C for two minutes. At 72°C, the last extension step was set for a single 7-minute cycle. Using a molecular size measurement of one hundred base pairs (from Bioneer, Korea), the PCR products were seen on a 1.5% agarose gel stained with red safe nucleic acid staining (Intron, Korea) [17].

**Molecular methods** **for identification of carbapenemase genes**

12.5 μl of GoTaq® Green Master Mix (2X), 1 μl of forward and reverse primers (Macrogen, Korea), 3 μl of bacterial DNA, 0.5 μl of MgCl_2_, and 7 μl of double distilled and deionized water made up a total volume of 25 μl. The following conditions for PCR cycling were employed: 30 cycles of one minute each at 95^o^C, one minute at 50 or 55^o^C, one minute at 72^o^C, and ten minutes at 72^o^C (as indicated by Table 2). The PCR product was analyzed using a 100 bp molecular size marker gel and detected using Red Safe Nucleic Acid Staining [16] (Intron, Korea).

| Gene Name | Primer | Sequence (5′–3′) | Product Size (bp) | Annealing Temp (°C) | Reference |
| --- | --- | --- | --- | --- | --- |
| *B. cepacia complex recA* | BCR1 | TGACCGCCGAGAAGAGCAA | 1043 | 56 | [17] |
|  | BCR2 | CTCTTCTTCGTCCATCGCCTC |  |  |  |
| *bla_KPC_* | Forward (F) | ATG TCA CTG TAT CGC CGT CT | 345 | 55 | [4] |
|  | Reverse (R) | TTT TCA GAG CCT TAC TGC CC |  |  |  |
| *bla_IMP_* | Forward (F) | TTGACACTCCATTTACDG | 139 | 50 | [4] |
|  | Reverse (R) | GATYGAGAATTAAGCCACYCT |  |  |  |
| *bla_GES_* | Forward (F) | ATGCGCTTCATTCACGCAC | 864 | 55 | [18] |
|  | Reverse (R) | TAATCAGTGAGGCACCTATCTC |  |  |  |

-	***The description of carbapenemase testing through both phenotypic and genotypic approaches is valuable, but further clarification is needed on how results were interpreted in case of discrepancies between the two methods. This would help readers understand the rationale behind the final conclusions on resistance.***

**Response to Reviewer:**

We appreciate your thoughtful comment. We recognize the significance of elucidating the methods used to resolve differences between genotypic and phenotypic carbapenemase detection. In order to better understand these results, we have included an explanation in the manuscript's Results section.

Both phenotypic and genotypic techniques were used in this investigation to evaluate the synthesis of carbapenemase by isolates of *B. cepacia* and *A. sobria*. Although there was a high level of consistency, a subset of isolates showed differences between the two methods. To be specific, some isolates showed phenotypic carbapenemase activity using the meropenem-EDTA combined disc synergy test, although PCR results for the targeted genes (*bla_KPC_, bla_IMP_,* and *bla_GES_*) were negative. On the other hand, other isolates have carbapenemase genes but showed little to no activity in the phenotypic test. The availability of resistance mechanisms unrelated to carbapenemase synthesis, such as efflux pumps or porin loss, or the existence of additional carbapenemase genes (such as *bla_NDM_* and *bla_VIM_*) not present in the current PCR panel could be the cause of these differences. Furthermore, false-negative PCR results could have been caused by mutations in primer-binding areas. Given these limitations, accurate carbapenem resistance detection requires the combination of phenotypic screening and molecular testing, especially in clinical settings where prompt and accurate monitoring of antibiotics is crucial.

***5.Discussion of Results:***

- ***The discussion regarding the resistance profiles, especially with respect to B. cepacia and A. sobria, could be expanded to address the clinical implications of these findings more comprehensively. For example, how do these resistance patterns correlate with patient outcomes or impact therapeutic decisions? Also, how do the findings compare with other studies in the region or globally in terms of therapeutic options?***

**Response to Reviewer:**

We appreciate your insightful feedback. To address the clinical significance of the identified resistance patterns for *A. sobria* and *B. cepacia*, we have included more information to the discussion section. To put our findings in perspective and emphasize the therapeutic difficulties and public health importance of these multidrug-resistant pathogens, we also included comparative analysis with regional and international studies. These additions, in our opinion, increase the findings' clinical significance and impact.

“The resistance profiles found in this investigation highlight important treatment challenges, especially for infections caused by *A. sobria* and *B. cepacia*. The high rates of resistance to routinely used β-lactam antibiotics, such as trimethoprim/sulfamethoxazole and ceftazidime, severely restrict the alternatives for empirical treatment. For B. cepacia, a bacterium frequently found in individuals with immunocompromised or cystic fibrosis, prompt and efficient antibiotic therapy is crucial to preventing negative effects. Although limited, the observed susceptibility to minocycline and meropenem may provide alternate therapeutic options; nonetheless, the emergence of resistance to these medicines (about 40 and 38.2%, respectively) emphasizes the critical need for susceptibility-guided therapy.”

“In ultimately, these resistance profiles have significant clinical implications. They need the use of combination or second-line treatments, which could be more expensive, hazardous, or less effective. Although direct associations with patient outcomes were outside the purview of this investigation, infections brought on by multidrug-resistant *B. cepacia* and *A. sobria* are probably linked to longer hospital admissions, higher morbidity, and maybe worse clinical outcomes. To better understand and control the burden of these new infections, future research that incorporates clinical outcome data will be crucial.”

- ***The prevalence of carbapenemase-producing strains is discussed, but the manuscript could benefit from more in-depth analysis on the mechanisms of carbapenem resistance. For instance, are there any particular resistance mechanisms (e.g., porin loss, overproduction of β-lactamases) that could explain the observed high levels of resistance in B. cepacia and A. sobria? The manuscript suggests that other resistance mechanisms could be at play, but this could be explored in more detail.***

**Response to Reviewer:**

We appreciate the reviewer's insightful observation that more information is needed to fully understand the mechanisms of carbapenem resistance than only the identification of the carbapenemase gene. To address alternative resistance mechanisms that might be responsible for the high levels of carbapenem resistance found in isolates of B. cepacia and A. sobria, we have extended the discussion section.

“Alternative processes, including as porin loss, efflux pump overexpression, and the overproduction of AmpC or extended-spectrum β-lactamases (ESBLs) in combination with decreased outer membrane permeability, can also lead to carbapenem resistance, as has been well documented in the literature [30]. When paired with β-lactamase synthesis, porin mutations or deletions decrease antibiotic inflow, which can result in high-level resistance even when carbapenemase genes are absent. In B. cepacia, which is renowned for having a wide range of resistance determinants and an inherently low outer membrane permeability, this is especially pertinent [21]. Furthermore, by actively extruding carbapenems and other β-lactams, efflux systems like the AcrAB-TolC pump and other resistance-nodulation-division (RND) family transporters may greatly increase resistance in A. sobria [17].”

***6.	Statistical Analysis:***

***While the study includes some breakdowns of resistance patterns (e.g., percentage of resistance), the manuscript lacks a detailed statistical analysis of the data. Including confidence intervals, significance testing (e.g., chi-square tests for categorical variables like gender and resistance patterns), and comparative analysis would improve the robustness of the findings.***

**Response to Reviewer:**

“The Chi-square test statistical analysis showed a significant association between isolates' carbapenem resistance and patient gender (χ² = 7.52, p = 0.006). In isolates from male patients, resistance was significantly higher (86.4%, 95% CI: 74.5–94.1%) than in those from female patients (58.1%, 95% CI: 39.1–75.5%). These results call for more research since they point to a possible gender-related difference in resistance acquisition or healthcare exposure”

“Of the 42 isolates of *B. cepacia*, 47.6% (20/42; 95% CI: 33.0%–62.6%) showed resistance to imipenem, and 66.7% (28/42; 95% CI: 51.6%–79.1%) showed resistance to meropenem. However, among the 14 isolates of *A. sobria*, 50.0% (7/14; 95% CI: 26.0%–74.0%) were resistant to imipenem, while 64.3% (9/14; 95% CI: 38.8%–83.7%) were resistant to meropenem. There was no significant correlation between species and meropenem resistance, according to a chi-square test (χ² = 0.169, p = 0.681), suggesting that the two species' resistance rates were comparable. Similarly, imipenem resistance and species did not show a statistically significant correlation (χ² = 0.034, p = 0.853), indicating similar resistance levels”

“The presence of carbapenemase genes (*bla_KPC_, bla_GES_*), as well as the lack of any carbapenemase gene, was significantly correlated with carbapenem resistance in isolates of *B. cepacia* and *A. sobria*, according to the Chi-square test for independence (χ² = 22.83, p =0.05). In particular, isolates with the *bla_KPC_* gene (90.5%, 95% CI: 85.1–95.0%) exhibited considerably higher resistance than isolates with the *bla_GES_* gene (66.7%, 95% CI: 41.3–88.3%) or without the carbapenemase gene (50.0%, 95% CI: 31.5–68.5%). These results underline the crucial role that carbapenemase genes play in resistance mechanisms by indicating a substantial correlation between their existence and carbapenem resistance”

**Minor Comments and Typos:**

1. **Typo/Grammar:**
   - ***"No A. sobria was found in any of the cerebrospinal fluid specimens." → "No A. sobria isolates were found in any of the cerebrospinal fluid specimens."***

**Response to Reviewer:**

I appreciate your observation. As indicated, the sentence has been clarified. It reads as follows: "No *A. sobria* isolates were found in any of the cerebrospinal fluid specimens." The amended version has been modified to reflect this adjustment, which enhances the statement's grammatical correctness.

o	"***This is consistent with Pineda-Reyes et al. (2024), who observed that A. sobri is linked to a variety of human infections..." → "A. sobria" (should match the species name format consistently).***

**Response to Reviewer:**

We appreciate you pointing out the inconsistent formatting of the species name. "A. sobri" has been changed to "*A. sobria*" to maintain uniformity with appropriate taxonomic names across the entirety of the manuscript. The updated version now includes this change.

***o	Please be consistent in naming species, especially with the genus and species names like B. cepacia and A. sobria. Use italics throughout and ensure the first letter of the genus is capitalized.***

**Response to Reviewer:**

We appreciate your attention to detail. We have thoroughly reviewed the manuscript and ensured that all bacterial species names, including B. cepacia and A. sobria, are consistently formatted in italics with the genus capitalized and the species name in lowercase, in accordance with standard taxonomic conventions.

***Ensure uniform citation formatting throughout the manuscript. There are a few inconsistencies in the way references are presented (e.g., reference numbering).***

**Response to Reviewer:**

Thank you for highlighting this important point. We have carefully reviewed and revised the manuscript to ensure uniform citation formatting throughout. All reference numbers have been checked for accuracy and consistency, and the citation style has been standardized in accordance with the journal’s guidelines.

This reference was removed. “Hussein RA, Al-Ouqaili MTS, Majeed YH. Association between alcohol consumption, cigarette smoking, and Helicobacter pylori infection in Iraqi patients submitted to gastrointestinal endoscopy. J Emerg Med Trauma Acute Care [Internet]. 2022 Dec 22 [cited 2022 Dec 23];2022(6):12. Available from: <https://www.qscience.com/content/journals/10.5339/jemtac.2022.aimco.12>”

**2.	Figures/Tables:**

o	***The manuscript could benefit from including figures or tables that summarize the major findings, such as the resistance rates for each antibiotic tested, the types of specimens sampled, and a comparison of resistance patterns between B. cepacia and A. sobria***

**Response to Reviewer:**

We value the reviewer's insightful recommendation to include graphic summaries. We would like to respond by pointing out that a figure comparing the resistance patterns of *A. sobria* and *B. cepacia* has been included to the revised manuscript. The resistance percentages for each tested drug are clearly shown in this image, making interpretation simpler and increasing the significance of our results. As advised, we have also provided a table that summarizes the resistance rates and the kinds of clinical specimens that were sampled.

**Figure 1. Patterns of drug resistance among the bacteria considered in the investigation**

**Response to Reviewer:**

We appreciate the reviewer’s recommendation to improve the clarity and accessibility of the data. In response, we have included Table 3 in the revised manuscript, which presents a detailed breakdown of the demographic characteristics (gender and age) and types of clinical samples from which the bacterial isolates were obtained. This table allows for a clearer understanding of the distribution of B. cepacia and A. sobria across different patient demographics and specimen types.

**Table 3. The distribution of study bacteria in relation to sample type, age, and gender of the subjects**

| Demographic Characteristics | Bacterial Isolates | | | | Total (n, %) |
| --- | --- | --- | --- | --- | --- |
|  | *B. cepacia* | | *A. sobria* | |  |
|  | n. | % | n. | % |  |
| Gender |  |  |  |  |  |
| Male | 38 | 50.7% | 6 | 8% | 44 (58.7%) |
| Female | 17 | 22.6% | 14 | 18.7% | 31 (41.3%) |
| Age (years) |  |  |  |  |  |
| ≥10 | 5 | 6.7% | 1 | 1.3 % | 6 (8%) |
| 11–20 | 1 | 5.3 % | 2 | 2.6 % | 3 (4 %) |
| 21–30 | 14 | 18.6 % | 8 | 10.7% | 22 (29.3%) |
| 31–40 | 9 | 12 % | 1 | 1.3 % | 10 (13.3%) |
| 41–50 | 1 | 1.3 % | 3 | 4 % | 4 (5.3 %) |
| 51–60 | 3 | 4 % | 1 | 1.3 % | 4 (5.3 %) |
| > 60 | 22 | 29.3 % | 4 | 5.3 % | 26 (34.6%) |
| Type of sample |  |  |  |  |  |
| Wound swab | 26 | 34.6% | 8 | 10.6% | 34 (45.3%) |
| Urine | 18 | 24 % | 7 | 9.3 % | 25 (33.3%) |
| Ear swab | 1 | 1.3 % | 1 | 1.3 % | 2 (2.6 %) |
| Cerebrospinal fluid | 2 | 2.6 % | 0 | 0 % | 2 (2.6 %) |
| Diabetic foot ulcer | 5 | 6.7% | 2 | 2.7 % | 7 (9.3%) |
| Blood | 3 | 4 % | 2 | 2.7 % | 5 (6.7%) |

***3.	Conclusion:***

***The conclusion is well-written but could be more focused on specific recommendations for future research and clinical practice. A few key research questions or clinical practice guidelines based on the findings would strengthen this section. For example, should clinicians be more cautious when using specific antibiotics for B. cepacia and A. sobria infections based on the resistance patterns observed?***

**Response to Reviewer:**

Thank you for your valuable feedback. We have made the necessary corrections and added the suggested explanations

Conclusion

According to this study, *B. cepacia* and *A. sobria* exhibit a high level of resistance to antibiotics, particularly to common β-lactam medications such as cefepime, piperacillin, and ceftriaxone. For infections involving these organisms, clinicians should exercise caution when selecting empirical antibiotic therapy, especially in high-risk patients. Instead, they ought to consider alternative treatments that are guided by susceptibility testing. As traditional phenotypic methods may not be precise enough to differentiate between various forms of carbapenemase, the prevalence of *bla_KPC_* and *bla_GES_* carbapenemase genes among the resistant isolates underscores the urgent need for routine molecular surveillance. This study shows that, from a diagnostic perspectives, the recA gene-based molecular approach provides an accurate and inexpensive alternative for the VITEK 2 system in recognizing *B. cepacia* complex members. PCR is still the best method for accurate carbapenemase gene detection because of its sensitivity and specificity. Whole-genome sequencing (WGS) of isolates that produce carbapenemase is recommended for future studies in order to determine their phylogenetic connects and potential for clonal propagation. Furthermore, more research should be done on *recA* nucleotide sequence analysis as a standardized technique for quickly identifying both new and old genomovars in the B. cepacia complex. This would improve patient treatment plans and infection control efforts by improving our understanding of each genomovar's clinical risks and pathogenic potential.

***There seems to be some inconsistency in the way data is reported. For example, you mention percentages in some cases but then refer to absolute numbers in others (e.g., "58.7% male" vs. "44 males"). It would be clearer and more standardized to report either both absolute numbers and percentages, or just one for consistency.***

**Response to Reviewer:**

Thank you for your observation. We have revised the manuscript to ensure consistency in data reporting by including both absolute numbers and corresponding percentages throughout the text.

***. Out of the 44, 38 (50.7%), B. cepacia detected in of male patients while A. sobria was detected in 6 (8%). With regard to the 31 (41.3%) female patients, B. cepacia was detected in 17 (22.6%) and A. sobria in 14 (18.7%).***

1. ***Lack of clarity about the sampling method and inclusion/exclusion criteria. Please clarify.***

**Response to Reviewer:**

**I appreciate your insightful comments. We have updated the manuscript to address your concern about the lack of clarity of the sampling procedure and inclusion/exclusion criteria. We have specifically specified the inclusion and exclusion criteria utilized in the study and provided clarification on the sampling process, including how participants were chosen.**

A total of 120 patients who attended the Ramadi Teaching Hospitals in the Al-Anbar Governorate between January and July 2024 were chosen at random to provide specimens. The participants were 50 women (41.7%) and 70 men (58.3%), with ages ranging from 10 to 70. Participants in the study were specifically chosen based on their age, clinical status, and the kind of specimens that were collected, in addition to additional inclusion criteria. All patients had an equal chance of being included, and bias was reduced by using the random sampling procedure.. A questionnaire was used to gather data on the included patients. It is crucial to carefully follow certain inclusion and exclusion criteria for isolating *B. cepacia* and A. sobria from clinical samples such cerebrospinal fluid (CSF), urine, ear swabs, burns, and wounds. Patients with underlying diseases such as cystic fibrosis or those with impaired immune systems should be included in the *B. cepacia* study, particularly if they exhibit symptoms like pneumonia, bacteremia, or infections of the central nervous system. Priority is given to samples from clinically relevant locations, such as wound cultures or CSF, while asymptomatic colonization or environmental contamination should be avoided. A. sobria is also isolated from samples that indicate active infections, such as urine, wound, and burn specimens, especially in immunocompromised people. Similar to B. cepacia, A. sobria should not be found in non-infectious or asymptomatic locations or in samples that might have environmental contamination
2. ***Justification for selecting only three carbapenemase genes (blaKPC, blaGES, blaIMP) is insufficient—why you exclude blaNDM, blaOXA, blaVIM Please give the reasons?***

**Response to Reviewer:**

The selection of **blaKPC**, **blaGES**, and **blaIMP** carbapenemase genes for this study was based on their higher prevalence and clinical significance in *B. cepacia* and *A. sobria* isolates, as well as the availability of reliable molecular detection methods. In contrast, **blaNDM**, **blaOXA**, and **blaVIM** were excluded due to their relatively low occurrence in these species, as well as the less frequent use of detection methods for these genes in routine clinical practice for *B. cepacia* and *A. sobria*. This focused approach allowed for a more comprehensive analysis of the most common and impactful resistance mechanisms in these isolates, in line with local prevalence data and clinical relevance.
3***. Only basic chi-square testing is used. A more robust analysis (e.g., multivariate regression) should be considered to correlate resistance genes with demographics or clinical variables.***

Response to Reviewer

Thank you for your valuable comment. Based on a statistician’s recommendation, we have confirmed that the chi-square test is appropriate for our categorical data. To strengthen the analysis, we have added 95% confidence intervals to key comparisons. This addition improves the interpretability and reliability of our findings, in line with best statistical practices. Relevant updates have been made to the methods and results sections of the manuscript.

***4. Figures (e.g., gel electrophoresis) are low resolution and poorly labeled.***

Response to Reviewer

Thank you for pointing this out. We have updated the figures, including the gel electrophoresis images, by replacing them with high-resolution versions and improving the labels, legends, and annotations to ensure clarity and readability. We believe these revisions enhance the visual quality and overall presentation of the data.

***5. Tables need improved formatting; define all abbreviations and ensure legends are self-explanatory.***

Response to Reviewer

I appreciate your helpful suggestions. All of the tables have been updated for better readability and formatting. We have revised the table legends to make sure they are completely self-explanatory and offer enough context without requiring reference to the main text, and we have provided explicit definitions for all abbreviations.

***6. Sample size reporting is occasionally inconsistent (e.g., 75 vs 56 isolates).***

Response to Reviewer

I appreciate you pointing out the constancy of sample size. To be clear, there were 75 isolates in our investigation, including 20 Aeromonas sobria and 55 Burkholderia cepacia. 56 of these 75 isolates (74.6%) had carbapenem (meropenem and imipenem) resistance, according to the VITEK-2 System. Of the 56 isolates that were resistant to carbapenem, 14 (25%) were *A. sobria* and 42 (75%) were *B. cepacia*.

We have revised the relevant sections of the manuscript to ensure consistent and accurate reporting of sample sizes and resistance proportions throughout.
***7.Several results are repeated multiple times across the text, tables, and figures.***

Response to Reviewer

Thank you for your helpful observation. We have carefully reviewed the manuscript and revised sections where results were unnecessarily repeated across the text, tables, and figures. To improve clarity and avoid redundancy, we have streamlined the presentation by summarizing key findings in the text while referring readers to the appropriate tables and figures for detailed data. These revisions enhance the manuscript’s readability and ensure a more concise and focused presentation of the results.

***8.Lack of MIC50/MIC90 or ranges diminishes the quality of antibiotic resistance interpretation.***

Response to Reviewer

According to design of our experiment we don’t need to detect MIC50/MIC90 because we need to detect the high level intermediate resistance of antimicrobial agents and detection of antibiotic resistance genes. Thank you for your observation

***9.The discussion is largely descriptive and lacks critical evaluation of findings.***

Response to Reviewer

Thank you for your valuable feedback. We have revised the Discussion section to move beyond descriptive reporting by providing a more critical evaluation of our findings. This includes comparing our results with previous studies, discussing possible mechanisms of resistance, and addressing the clinical implications and limitations of our data. These revisions aim to enhance the depth and analytical quality of the discussion.

***10. Clinical implications of findings are weakly addressed (e.g., impact on treatment policy or infection control practices).***

Response to Reviewer

Thank you for your insightful comment. We have revised the Discussion section to include the clinical implications of our findings, particularly regarding their potential impact on treatment policies and infection control practices.

“In ultimately, these resistance profiles have significant clinical implications. They need the use of combination or second-line treatments, which could be more expensive, hazardous, or less effective. Although direct associations with patient outcomes were outside the purview of this investigation, infections brought on by multidrug-resistant *B. cepacia* and *A. sobria* are probably linked to longer hospital admissions, higher morbidity, and maybe worse clinical outcomes. To better understand and control the burden of these new infections, future research that incorporates clinical outcome data will be crucial. “

***11. Mentions “genomic sequencing” without this being part of the study.***

Response to Reviewer

Thank you for your observation. We acknowledge that genomic sequencing was not conducted in the current study. We have clarified this point and now refer to genomic sequencing in the Conclusion section as a recommendation for future research to further characterize resistance mechanisms and genetic determinants.

***12.Inconsistent use of scientific names (italicization of B. cepacia, A. sobria must be needed throughout the manuscript).***

Response to Reviewer

Thank you for pointing this out. We have carefully reviewed the manuscript and corrected all instances of scientific names, including B. cepacia and A. sobria, to ensure consistent italicization in accordance with scientific conventions throughout the revised manuscript.

***13. Abstract needs grammatical and syntactical revisions (e.g., “92.8%%” should be “92.8%”).***

Response to Reviewer

Thank you for your helpful comment. We have thoroughly revised the abstract to correct all grammatical and syntactical errors, including typographical issues such as “92.8%%,” which has been corrected to “92.8%.”
14 Some references are outdated, and certain statements require more recent supporting evidence.

***15. In methodology: Study design (cross-sectional) and sampling methods are acceptable but Was the sampling random or convenience-based? What were the inclusion/exclusion criteria?Ethical approval and consent are reported appropriately. Primer sequences and thermocycling protocols should be summarized more clearly in one table. Justification for excluding genes like blaNDM, blaOXA, or blaVIM is missing. The phenotypic test (double disk synergy) is correctly described but lacks proper validation or controls explanation.***

Response to Reviewer

Thank you for your helpful comment.The study employed a cross-sectional design with random sampling, which is appropriate for estimating the prevalence of characteristics or outcomes in a population. Thank you for your suggestion. We have revised the manuscript and now present the primer sequences and thermocycling protocols in a single, clearly organized table for better clarity and accessibility.

| Gene Name | Primer | Sequence (5′–3′) | Product Size (bp) | Annealing Temp (°C) | Reference |
| --- | --- | --- | --- | --- | --- |
| *B. cepacia complex recA* | BCR1 | TGACCGCCGAGAAGAGCAA | 1043 | 56 | [17] |
|  | BCR2 | CTCTTCTTCGTCCATCGCCTC |  |  |  |
| *bla_KPC_* | Forward (F) | ATG TCA CTG TAT CGC CGT CT | 345 | 55 | [4] |
|  | Reverse (R) | TTT TCA GAG CCT TAC TGC CC |  |  |  |
| *bla_IMP_* | Forward (F) | TTGACACTCCATTTACDG | 139 | 50 | [4] |
|  | Reverse (R) | GATYGAGAATTAAGCCACYCT |  |  |  |
| *bla_GES_* | Forward (F) | ATGCGCTTCATTCACGCAC | 864 | 55 | [18] |
|  | Reverse (R) | TAATCAGTGAGGCACCTATCTC |  |  |  |

***16. There is confusion in sample size reporting: The abstract mentions 75 isolates, with 20 A. sobria and 55 B. cepacia, totaling 75, yet elsewhere “56 carbapenem-resistant isolates” are analyzed without clear criteria. Please give Justification??***

Response to Reviewer

I appreciate you pointing out the constancy of sample size. To be clear, there were 75 isolates in our investigation, including 20 Aeromonas sobria and 55 Burkholderia cepacia. 56 of these 75 isolates (74.6%) had carbapenem (meropenem and imipenem) resistance, according to the VITEK-2 System. Of the 56 isolates that were resistant to carbapenem, 14 (25%) were *A. sobria* and 42 (75%) were *B. cepacia*.

We have revised the relevant sections of the manuscript to ensure consistent and accurate reporting of sample sizes and resistance proportions throughout.

***17. Use of vague terms like “high”, “little resistance”, or “more accurate” should be replaced with quantified expressions.***

Response to Reviewer:

Thank you for your valuable feedback. In response, we have replaced vague terms such as “high,” “little resistance,” and “more accurate” with more quantified expressions to provide clearer and more precise descriptions of our findings. These revisions enhance the accuracy and clarity of the manuscript.

***18. Not all abbreviations are defined e.g., MEM, IMP are missing.***

**Response to Reviewer:**

Thank you for your observation. We have reviewed the manuscript and ensured that all abbreviations, including **MEM** (meropenem) and **IMP** (imipenem), are clearly defined the first time they appear in the text. The revised manuscript now includes these definitions for improved clarity.

***19.Some values are redundant across tables and figures.***

**Response to Reviewer:**

Thank you for your helpful feedback. We have reviewed the manuscript and **removed redundant values** across tables and figures to avoid repetition.

***20.The absence of sequencing data for PCR amplicons is a major limitation. Without sequencing, false-positive bands from non-specific amplification cannot be ruled out. Lack of controls (positive and negative) for each PCR gene detection is not mentioned.***

**Response to Reviewer:**

Thank you for highlighting this important point. The confirmation of specific PCR band by was confirmed by the presence of DNA ladder and comparison the size (bp) of DNA ladder with the those of positive band. The nonspecific band ( at size other than DNA ladder were excluded from our study). We have also noted that sequencing of PCR amplicons is planned for future studies to validate the findings and rule out non-specific amplification.

***21. According to CLSI guidelines, the double-disk synergy test (DDST) is not the gold standard for carbapenemase detection; tests like Carba NP or modified Hodge test should be used.***

**Response to Reviewer:**

Thank you for your valuable comment. We acknowledge that, according to CLSI guidelines, methods such as the Carba NP test or modified Hodge test are more widely recognized as confirmatory tests for carbapenemase production. However, we selected the Double-Disk Synergy Test (DDST) due to its cost-effectiveness, ease of use, and suitability in resource-limited settings, where access to molecular or advanced phenotypic tests may be restricted. Nonetheless, we acknowledge this as a methodological limitation and have included a statement in the revised Discussion section suggesting the use of more specific confirmatory tests, such as Carba NP, in future studies.

***22. The study do not correlate clinical outcomes (e.g., mortality, hospital stay) with resistance profiles. Host factors, such as patient comorbidities, prior antibiotic exposure, and ICU admission, are not considered in the study. Please consdider for these factors.***

**Response to Reviewer**

Thank you for your insightful comment. We acknowledge the importance of correlating resistance profiles with clinical outcomes and host factors such as comorbidities, prior antibiotic use, and ICU admission. However, the primary aim of our study was to focus on the detection of antibiotic resistance patterns and the identification of resistance genes in the isolates. While clinical correlations were beyond the scope of this investigation, we agree they are valuable and recommend that future studies incorporate these factors to provide a more comprehensive understanding of the clinical impact of antimicrobial resistance.

***23.The authors state adherence to the “Helsinki Declaration of 1979,” which is outdated; the 2013 version is recent Helsinki Declaration.***

**Response to Reviewer**

We appreciate your feedback. The manuscript has been updated to comply with the latest version of the Declaration of Helsinki, specifically the 2013 revision, ensuring both accuracy and adherence to current ethical standards.
