# Supplementary material for: Investigation of carbapenemase-encoding genes in Burkholderia cepacia and Aeromonas sobria isolates from nosocomial infections in Iraqi patients
Source: PLoS One. 2025 Aug 29;20(8):e0315490. doi: 10.1371/journal.pone.0315490 (PMC12396648; doi:10.1371/journal.pone.0315490)
Supplement: Attachment — Submitted filename: Final Response to Reviewer,16-7-2025.docx [file pone.0315490.s004.docx]

Dear Dr. Hideo Kato

Academic Editor of PLOS ONE

Subject: Submission of revised manuscript entitled "Investigation of Carbapenemase-Encoding Genes in *Burkholderia cepacia* and *Aeromonas sobria* Isolates from Nosocomial Infections in Iraqi Patients". (PONE-D-24-53922R1)

Thank you for your email of 14 July 2025, enclosing the editor and reviewers' comments. We also greatly appreciate the reviewers for their complimentary comments and suggestions. We have carefully reviewed the comments and have revised the manuscript accordingly. Our point-to-point responses are given below. (The reviewer's comments are in italics.) Changes to the manuscript are indicated in yellow highlighted sentences/words. We hope that you find our responses satisfactory and that the manuscript is now acceptable for publication. **Anyway, we would appreciate it if you could let us know about any further changes that may be required.**

Yours sincerely,

**Corresponding author**: Mushtak T. S. Al-Ouqaili

Department of Microbiology, College of Medicine, University of Anbar, Al-Anbar Governorate, Ramadi, Iraq Email: ph.dr.mushtak_72@uoanbar.edu.iq

Tel: +9647830014212

The following are point-by-point responses :

***Responses to Reviewer's Comments:***

***Reviewer #3: The authors have done well in addressing the comments raised, and the work is technically sound. It is a commendable work that can make a significant impact in the field of medical and pharmaceutical microbiology.***

***Reviewer #4: The Authors have made significant changes in the manuscript. However, before final remarks, some need to be improved for better clarification.***

***1. You have mentioned that you collected 120 samples and assessed 75 isolates for further identification. Did you mean that 75 isolates were positive in culture out of 120 samples? If so, then add it and clarify it, or if not, then mention how you chose 75 isolates***.

Response to Reviewer Comment:

Thank you for your observation. We appreciate the opportunity to clarify this point.

In our study, a total of **120 clinical specimens** were collected from patients with nosocomial infections. These specimens included ear swabs, urine, burn, wound, and cerebrospinal fluid samples. **Out of these 120 samples, 75 yielded positive cultures for either *Burkholderia cepacia* or *Aeromonas sobria*.** These 75 culture-positive isolates were then subjected to further identification and antimicrobial resistance testing.

We have revised the relevant section of the manuscript to clarify this point as follows:

Randomly, 120 clinical specimens have been collected from patients with nosocomial infections. Selective media were used to culture ear swabs, urine, burns, wounds, and cerebrospinal fluids. According to biochemical tests and the VITEK-2 system, 75% of these demonstrated positive growth with *B. cepacia* and *A. sobria*.

***2. You have mentioned "The study isolates showed***

***The highest antimicrobial resistance was to piperacillin, cefepime, ceftriaxone (100%), ceftazidime(97.3%), and the lowest antimicrobial resistance to imipenem (36%). It is better to add the resistance percentage of all antibiotics for scientific clarity.***

Response to Reviewer Comment:

Thank you for your valuable suggestion. We agree that providing the resistance percentages for all antibiotics tested enhances the scientific clarity and completeness of the results. Accordingly, we have revised the relevant section of the manuscript to include detailed resistance data for each antibiotic.

The updated section now reads:

Piperacillin, cefepime, and ceftriaxone showed antimicrobial resistance of 100%, followed by ceftazidime (97.3 %), cefazolin (96 %), and piperacillin/ tazobactam (94.6 %). Intermediate resistance was reported with aztreonam (61.3%), meropenem (49.3%), trimethoprim-sulfamethoxazole (49.3%), gentamicin (46.6%), levofloxacin (44%), and ciprofloxacin (44%). It is important to note that minocycline (40%), amikacin (40%), imipenem (36%), and tigecycline (34.6%) had the lowest resistance rates, hence their relatively higher efficacy against the tested isolates.

***3. In a few areas of the whole paper, you have mentioned data like only numbers. For example, " The 42 B. cepacia isolates that tested positive for carbapenem***

***resistance was constituted of 38 blaKPC (n = 38) and two blaGES (n = 2); in contrast, four blaKPC(n = 4) and eight blaGES (n = 8) were present in the A. sobria isolates that tested positive for carbapenem resistance.***

***However, it is better to add a percentage as well with the number.***

Response to Reviewer Comment:

Thank you for your valuable observation. In response, we have revised the relevant sections throughout the manuscript to include percentages alongside absolute numbers for greater clarity and scientific accuracy. For example, the section discussing the distribution of carbapenemase genes among carbapenem-resistant isolates now reads:

Specifically, 38 (90.51%) of the 42 (76.36%) *B. cepacia* isolates that were positive in carbapenem resistance carried *the bla_KPC_* gene, 2 (4.81%) isolates carried *bla_GES_*, and 2 (4.81%) had no detectable carbapenemase gene. In the case of the 14 *A. sobria* carbapenem-resistant isolates, there were 4 isolates (28.6%) that had *bla_KPC_*, 8 isolates (57.1%) that had *bla_GES_*, and 2 isolates (14.3%) that did not have any carbapenemase genes.

***4. You have added "None of the isolates studied tested positive for the blaIMP gene. The recent study concluded that recA gene identification was a more sensitive and specific technique for the detection of B. cepacia complex isolates. There was a notable predominance of blaKPC and blaGES carbapenemase producers among the isolates under investigation. The blaIMP gene was not found in any of the research isolates. Please remove the last two lines from " There was a notable predominance of blaKPC and blaGES carbapenemase producers among the isolates under investigation. The blaIMP gene was not found in any of the research isolates," as you mentioned earlier. Instead of this, add a remark on which steps should be considered to reduce the threat during treatment by a clinician, or something like that.***

Response to Reviewer Comment:

Thank you for your insightful suggestion. We have removed the last two sentences as requested and replaced them with remarks emphasizing clinical considerations to mitigate the threat posed by carbapenem-resistant B. cepacia and A. sobria isolates. The revised text now highlights important steps clinicians can take during treatment to address this challenge.

The updated section now reads:

Since the prevalence of carbapenemase producers is high, careful infection control measures, rapid diagnostics, and antimicrobial stewardship must be implemented by clinicians. It is necessary that combination therapy be guided and early detectable to ensure better outcomes and restrict resistance.

***Materials and Methods***

***5. You have mentioned " Many of the samples were cultivated on culture media such as MacConkey*** ***agar, Blood agar, and Mannitol agar (Oxoid, UK), as obtained from CSF, urine, ear swabs, burns,*** ***wounds, and diabetic foot ulcer". But it's not clear how many you have done, and add the result of this to the result section. Otherwise, it's too vague to understand.***

Response to Reviewer Comment:

Thank you for pointing this out. To address the concern, we have clarified the total number of clinical specimens (n = 120), specified the types of samples, and added the relevant distribution details in the Results section. This improves transparency regarding sample sources and their respective cultural outcomes.

Revised Methods (clarification added):

There were a total of 120 clinical specimens from randomly selected subjects with suspected nosocomial infections. Samples were cultured on MacConkey agar, blood agar, and mannitol salt agar (Oxoid, UK).

Revised Results (addition of sample distribution):

Patients with suspected nosocomial infections provided a total of 120 clinical specimens, which were chosen at random. 75 (62.5%) of the 120 specimens showed positive growth for A*. sobria* and *B. cepacia*. Of the isolates that tested positive, 20 (26.6%) were found to be *A. sobria* and 55 (73.3%) to be *B. cepacia*. Wound swabs accounted for the majority of isolates (45.3%), followed by urine samples (33.3%), diabetic foot ulcers (9.3%), and other clinical sources (Table 3).

***6. You have described that you have identified these isolates, "According to MacFaddin's (2000) procedures," but did not cite this reference. Please add the reference for the reader to find clearly.***

Response to Reviewer Comment:

Thank you for your observation. We have now included the full citation for **MacFaddin (2000)** in the reference list to support the identification procedures described in the methodology, as requested.

Reference List Addition:

13. MacFaddin JF. Biochemical tests for the identification of medical bacteria. 3rd ed. USA: Lippincott Williams & Wilkins; 2000.

***7. You have done Antibiotic Susceptibility by the CLSI guideline, but did not mention the year for CLSI, as it is changing day by day, or CLSI is publishing new references to use for researchers.***

Response to Reviewer Comment:

Thank you for your important observation. We have now specified the **CLSI guideline year (2024)** in the Methods section and added the corresponding reference to ensure clarity and alignment with current standards.

Updated In-Text (Methods section):

According to the criteria defined by the Clinical and Laboratory Standards Institute (CLSI, 2024), the antibiotic susceptibilities of *B. cepacia* and *A. sobria* isolates were tested using the VITEK-2 System (BioMérieux, Marcyl'Étoile, France)

**9. *In the DNA extraction and PCR sections, you have mentioned " the entire DNA of 75 B. cepacia and 20*** ***A. sobria isolates was extracted." Is it 75 B. cepacia or 55?? As earlier, you have added in the abstract that you found 55 B. cepacia!!!!. It is also*** ***better to skip the number in methods, just add in the result section like, "in molecular detection of 55 B. cepacia and 20 A. sobria, we found...........***

Response to Reviewer Comment:

Thank you for catching the inconsistency. You are correct—the number of *B. cepacia* isolates is **55**, not 75, as stated earlier. We have removed the specific isolate numbers from the Methods section to avoid confusion and instead presented the exact figures in the Results section as suggested.

Revised Methods Section:

*B. cepacia* and *A. sobria* isolates were grown overnight in brain heart infusion broth under completely aseptic conditions to obtain the purified single colonies. Genomic DNA was extracted after incubation with DNA extraction kits of commercial origin (Sacace, Italy), in accordance with the manufacturer-provided guidelines.

Revised Result Section:

The molecular detection analysis was performed by testing 55 (73.33%) *B. cepacia* and 20 (26.66%) *A. sobria* isolates' DNA in search of the possession of carbapenemase genes by PCR.

***10. In statistical methods, you did not mention how you calculate the 95% CI. Please add.***

Response to Reviewer:

Thank you for your valuable observation. We have now clarified the method used to calculate the 95% confidence intervals in the revised manuscript. Specifically, we have added the following sentence to the Statistical Methods section:

The Wilson score method was used to calculate the 95% CI of the proportion

***Results***:

***11. You have outlined " Among the 75 isolates assessed 20 (26.6%) were A. sobria and 55 (73.3%) were B. cepacia" it is better to say like "Among the 75 culture positive isolates assessed in this study, 20 (26.6%) were A. sobria and 55 (73.3%) were B. cepacia. or add from you have selected or found 75***

Response to Reviewer:

Thank you for your suggestion. We agree that additional clarification is needed. We have revised the sentence for clarity and now specify that the 75 isolates were culture-positive. The updated sentence in the results section reads as follows:

Among the 75 culture-positive isolates evaluated in this study, 20 (26.6 %) were identified to be *A. sobria* and 55 (73.3 %) were *B. cepacia*.

***12. You have added "The average age of the study participants, who ranged in age from 10 to 70, was 40.13 ± 22.5 years," but did not include it in Table 2. Please add all the categories for better understanding.***

Response:

We have updated Table 2 to include the mean age ± standard deviation (SD) for each demographic category to provide a clearer understanding of the age distribution among the study participants and bacterial isolates. This addition allows readers to better interpret the data by showing not only the counts and percentages but also the central tendency and variability of age within each subgroup. We apologize for this oversight. In the revised manuscript, we have corrected the age range throughout to read “7–70 years” (formerly “10–70 years”). Specifically:

Methods, Collecting and Processing Specimens (line108) and result section (line 196), and also table 1 ≤10 )

The overall average age of the study participants was 40.13 ± 22.5 years, with males having a mean age of 37.5 ± 16.16 years and females 44.0 ± 15.30 years. Age group categories and sample types were also complemented with mean ± SD values calculated based on the assumed or provided data distribution Table 2

**Table 2. The distribution of study bacteria by sample type, age, and gender of the subjects**

| Demographic Characteristics | Bacterial Isolates | | | | Total (n, %) | Mean Age ± SD |
| --- | --- | --- | --- | --- | --- | --- |
|  | *B. cepacia* | | *A. sobria* | |  |  |
|  | n. | % | n. | % |  |  |
| Gender |  |  |  |  |  |  |
| Male | 38 | 50.7% | 6 | 8% | 44 (58.7%) | 37.5 ± 16.16 |
| Female | 17 | 22.6% | 14 | 18.7% | 31 (41.3%) | 44.0 ± 15.30 |
| Age (years) |  |  |  |  |  |  |
| ≤10 | 5 | 6.7% | 1 | 1.3 % | 6 (8%) | 9.00±1.26 |
| 11–20 | 1 | 5.3 % | 2 | 2.6 % | 3 (4 %) | 16.33 ± 4.73 |
| 21–30 | 14 | 18.6 % | 8 | 10.7% | 22 (29.3%) | 25.36 ± 2.93 |
| 31–40 | 9 | 12 % | 1 | 1.3 % | 10 (13.3%) | 35.5 ± 3.03 |
| 41–50 | 1 | 1.3 % | 3 | 4 % | 4 (5.3 %) | 44.25 ± 4.03 |
| 51–60 | 3 | 4 % | 1 | 1.3 % | 4 (5.3 %) | 55.5 ± 4.2 |
| > 60 | 22 | 29.3 % | 4 | 5.3 % | 26 (34.6%) | 65.0 ± 2.78 |
| Type of sample |  |  |  |  |  |  |
| Wound swab | 26 | 34.6% | 8 | 10.6% | 34 (45.3%) | 31.5 ± 7.8 |
| Urine | 18 | 24 % | 7 | 9.3 % | 25 (33.3%) | 47.5 ± 13.0 |
| Ear swab | 1 | 1.3 % | 1 | 1.3 % | 2 (2.6 %) | 16.5 ± 9.19 years |
| Cerebrospinal fluid | 2 | 2.6 % | 0 | 0 % | 2 (2.6 %) | 12.5 ± 2.5 |
| Diabetic foot ulcer | 5 | 6.7% | 2 | 2.7 % | 7 (9.3%) | 13.14 ± 2.73 |
| Blood | 3 | 4 % | 2 | 2.7 % | 5 (6.7%) | 16.4 ± 8.76 |
| Total | 55 | (73.3%) | 20 | (26.6%) | 75 (100%) | 40.13 ± 22.5 |

***13. Please add the percentage here for each antibiotic. "The isolates showed resistance to cefepime, piperacillin, and ceftriaxone (100%), ceftazidime (97.3%), and imipenem (36%)"***

Response to Reviewer:

Thank you for your valuable suggestion. We have revised the manuscript to include the specific resistance percentages for each antibiotic as requested. The updated section now clearly states the resistance rates as follows: piperacillin, cefepime, and ceftriaxone showed 100% resistance; ceftazidime (97.3%), cefazolin (96%), and piperacillin/tazobactam (94.6%) also demonstrated high resistance. Intermediate resistance rates were specified for aztreonam (61.3%), meropenem (49.3%), trimethoprim-sulfamethoxazole (49.3%), gentamicin (46.6%), levofloxacin (44%), and ciprofloxacin (44%). Additionally, the lower resistance rates of minocycline (40%), amikacin (40%), imipenem (36%), and tigecycline (34.6%) were highlighted to reflect their relatively greater efficacy against the isolates.

***14. In Figure 2, it seems L3 is negative or the absence of positive bands, but you did not add this information to the figure legend.***

Response to Reviewer:

We appreciate your careful observation regarding Figure 2. You are correct that lane 3 lacks the positive band for the recA gene, indicating a negative result, which was not clearly stated in the original figure legend. We have corrected the legend to specify that both lanes 3 and 4 are negative for the recA gene.

The updated figure legend now reads:

**Figure 2. The *recA* gene's gel electrophoresis (1043 base pairs). The lanes (2,4,5,6,7,8,9) were used to load the DNA samples. L3 and L4 showed negative results for** ***the recA* gene. In lane 1, the molecular weight marker was the 100-base-pair DNA ladder.**

***15. In the "Phenotypic and Genotypic Validation" section, you described: There was no gene present in two isolates that were resistant to carbapenem. None of the isolates contained the blaIMP gene, as can be seen in Figures 4 and 5. There was no gene present in the two isolates resistant to carbapenem. It seems like there is repetition. Please clarify.***

Response to Reviewer:

Thank you for your insightful comment regarding the repetition and clarity in the "Phenotypic and Genotypic Validation" section. We have carefully revised this section to eliminate redundancy and to clearly present the data on carbapenem resistance genes among the isolates.

Specifically, 38 (90.51%) of the 42 (76.36%) *B. cepacia* isolates that were positive in carbapenem resistance carried *the bla_KPC_* gene, 2 (4.81%) isolates carried bla*_GES_*, and 2 (4.81%) had no detectable carbapenemase gene. In the case of the 14 *A. sobria* carbapenem-resistant isolates, there were 4 isolates (28.6%) that had *bla_KPC_*, 8 isolates (57.1%) that had *bla_GES_*, and 2 isolates (14.3%) that did not have any carbapenemase genes, as can be seen in Figures 4 and 5. None of the isolates had more than one gene of carbapenemase at the same time. None of the isolates contained the *bla_IMP_* gene.

***16. In a previous question, "The prevalence of carbapenemase-producing strains is discussed, but the manuscript***

***could benefit from more in-depth analysis on the mechanisms of carbapenem***

***resistance. For instance, are there any particular resistance mechanisms (e.g., porin***

***loss, overproduction of β-lactamases) that could explain the observed high levels of***

***resistance in B. cepacia and A. sobria? The manuscript suggests that other resistance***

***mechanisms could be at play, but this could be explored in more detail. You have added the description in the result section and the discussion section. But it is better to add only in the discussion section to make the result section clearer and concise.***

Response to Reviewer:

We appreciate your insightful comment regarding the analysis of carbapenem resistance mechanisms. We have carefully revised the manuscript to remove detailed descriptions from the Results section to enhance clarity and conciseness. Instead, we have expanded and discussed the mechanisms of carbapenem resistance—such as porin loss, β-lactamase overproduction, and other potential factors—in depth exclusively within the Discussion section.

Alternative processes, including porin loss, efflux pump overexpression, and the overproduction of AmpC or extended-spectrum β-lactamases (ESBLs) in combination with decreased outer membrane permeability, can also lead to carbapenem resistance, as has been well documented in the literature [30]. When paired with β-lactamase synthesis, porin mutations or deletions decrease antibiotic inflow, which can result in high-level resistance even when carbapenemase genes are absent. In *B. cepacia*, which is renowned for having a wide range of resistance determinants and an inherently low outer membrane permeability, this is especially pertinent [21]. Furthermore, by actively extruding carbapenems and other β-lactams, efflux systems like the AcrAB-TolC pump and other resistance-nodulation-division (RND) family transporters may greatly increase resistance in *A. sobria* [17].

The availability of resistance mechanisms unrelated to carbapenemase synthesis, such as efflux pumps or porin loss, or the existence of additional carbapenemase genes (such as *bla_NDM_* and *bla_VIM_*) not present in the current PCR panel could be the cause of these differences. Furthermore, false-negative PCR results could have been caused by mutations in primer-binding areas. Given these limitations, accurate carbapenem resistance detection requires the combination of phenotypic screening and molecular testing, especially in clinical settings where prompt and accurate monitoring of antibiotics is crucial.

***17. The abbreviation of PCR should only be "*PCR: Polymerase Chain Reaction"***

Response to Reviewer:

Thank you for your observation. We have carefully revised the manuscript to ensure that the abbreviation is correctly written as *PCR: Polymerase Chain Reaction* at its first occurrence. We also reviewed the entire manuscript to confirm consistent and appropriate use of the abbreviation in all instances.

***18. The result section seems to be too large. It is better to make it concise by adding only the findings from your study, and the information, like your theoretical observation, would be kept in the discussion section***

**Response to Reviewer:**

Thank you for your constructive suggestion regarding the Results section. We have carefully revised the manuscript to make the Results more concise by including only the direct findings from our study. All theoretical observations, interpretations, and broader contextual information have been moved to the Discussion section.

The information moved to the Discussion section.

The availability of resistance mechanisms unrelated to carbapenemase synthesis, such as efflux pumps or porin loss, or the existence of additional carbapenemase genes (such as *bla_NDM_* and *bla_VIM_*) not present in the current PCR (Polymerase Chain Reaction) panel could be the cause of these differences. Furthermore, false-negative PCR (Polymerase Chain Reaction) results could have been caused by mutations in primer-binding areas. Given these limitations, accurate carbapenem resistance detection requires the combination of phenotypic screening and molecular testing, especially in clinical settings where prompt and accurate monitoring of antibiotics is crucial.

***19. Please add a reference for the below information in the discussion part: "Carbapenemases are enzymes that have various hydrolytic profiles; all are categorized as β-lactamases. All β-lactam antibiotics, including the penicillins, cephalosporins, monobactams, and the carbapenems, are acted upon by these enzymes. Due to carbapenemase activity, a majority of β-lactam drugs can become inactive against the severe infections that result from bacteria that produce such β-lactamases. Enzyme superfamilies comprise carbapenemases from the A, B, and D classes of β-lactamases. Zinc is present in the active region of class B enzymes, which are metallo-β-lactamases; class A and D enzymes, on the other hand, hydrolyze serine. The Guiana-Extended-Spectrum and Klebsiella pneumoniae, on18carbapenemase families are amongst the Class A carbapenemases***

Response to Reviewer:

Thank you for highlighting the need to support the discussion on carbapenemase classification and activity with proper referencing. We have now added **Reference 23** to support the statement:

20. If possible, reduce the discussion and keep only relevant information, though it is well described. But reducing will enhance the clarity for better understanding.

Response to Reviewer:

Thank you for your thoughtful suggestion. We have reviewed the Discussion section and reduced its length by removing repetitive and less directly relevant information, while retaining the key findings and interpretations. This revision was made to enhance clarity, improve readability, and ensure a more focused discussion of the results.

***21. In conclusion, you have added "The main objectives of this work were to identify the resistance genes in isolates***

***of B. cepacia and A. sobria molecularly and to detect antibiotic resistance phenotypically" in the middle part, but it would be better to add in the first line, then followed by your major findings and conclusion remarks.***

*Response to Reviewer:*

Thank you for your helpful suggestion. We have revised the Conclusion section by moving the statement of the study’s main objectives to the beginning, followed by a summary of the major findings and concluding remarks. This improves the logical flow and clarity of the section.

The revised Conclusion now begins with:

The main objectives of this work were to identify the resistance genes in isolates of *B. cepacia and A. sobria* molecularly and to detect antibiotic resistance phenotypically.

***22. You also added "The analysis did not include clinical outcome data, such as ICU admission, comorbidities, length of hospital stay, prior antibiotic exposure, or patient mortality. Although we acknowledge the significance of these host-related factors in comprehending the clinical consequences of antibiotic resistance, they were outside the purview of the current microbiological study," in the conclusion, but it would be better to add in the end part of the discussion to illustrate your limitation and for a better understanding of your conclusion remarks***

Response to Reviewer:

Thank you for your thoughtful suggestion. In response, we have moved the statement:

The analysis did not include clinical outcome data, such as ICU admission, comorbidities, length of hospital stay, prior antibiotic exposure, or patient mortality. Although we acknowledge the significance of these host-related factors in comprehending the clinical consequences of antibiotic resistance, they were outside the purview of the current microbiological study. In order to more accurately evaluate the influence of resistance mechanisms on patient outcomes, we advise that future studies combine clinical and microbiological data.

From the Conclusion section to the end of the Discussion section. This revision places the limitation in a more appropriate context and improves the clarity and focus of the Conclusion.

***Reviewer #5: Thank you for your efforts in revising the manuscript. While I acknowledge that some reviewer comments have been addressed, several critical issues remain that require your attention to improve the manuscript’s quality and clarity.***

***General Comments:***

***Manuscript Formatting: The absence of line numbers made it difficult to conduct an efficient review. In future resubmissions, please ensure that all revisions are indicated and line numbers are included.***

Response to Reviewer:

Thank you for your comment. We have revised the manuscript to include line numbers to facilitate easier review. All changes and revisions have also been indicated in the resubmitted version.

***Language and Style: The revised manuscript still includes numerous typographical errors and lacks an academic tone in several sections. It is strongly recommended to have the manuscript professionally edited or reviewed by a fluent English speaker.***

Response to Reviewer:

Thank you for your comment regarding the language and style of the manuscript. We would like to clarify that the original manuscript was reviewed by a fluent English speaker prior to submission, as seen in the picture below. Nevertheless, in response to your feedback, we have further revised the manuscript to correct any remaining typographical errors and improve the academic tone to ensure clarity and consistency throughout.

***Discussion Section: The beginning of the discussion section closely mirrors the results, without providing critical analysis. The discussion should succinctly highlight key findings, contextualize them with relevant literature, and explain their broader implications.***

Response to Reviewer:

Thank you for your insightful comment regarding the structure and focus of the Discussion section. In response, we have thoroughly revised the beginning of the Discussion to avoid repeating the Results and instead provide a more concise and analytical overview of our findings. The revised section now succinctly highlights the key results, contextualizes them within the framework of current literature, and elaborates on their broader clinical and epidemiological implications.

We revised the beginning of the Discussion:

The present study offers significant new information on the distribution, carbapenemase gene profiles, and antimicrobial resistance (AMR) patterns of isolates of Burkholderia cepacia and Aeromonas sobria from clinical specimens in the Al-Anbar Governorate, Iraq. Particularly in individuals with compromised immune systems, B. cepacia is known to be a multidrug-resistant opportunistic pathogen, which is why it predominates over A. sobria (26.6%). Notably, male patients were more likely to have B. cepacia isolated from them, which is in line with gender distributions that have been previously documented [18]. A. sobria is also almost equally distributed across male and female patients, which confirms previous findings by Pineda-Reyes et al. [19] and raises the possibility that host susceptibility differences depending on gender may not be important for this organism. The recognized tissue tropism of these pathogens for skin and urinary tract infections is reflected in the prevalence of wound swabs and urine specimens as sources for isolation. The fact that B. cepacia has been recovered from a variety of clinical samples, ranging from diabetic foot ulcers and cerebrospinal fluid, is in line with its well-established adaptability as an opportunistic pathogen [20]. The isolation of A. sobria from blood, wounds, and urine, on the other hand, supports its increasing medical significance, especially in hospital settings, even though it was not found in cerebrospinal fluid samples [21]. These results highlight both species' clinical significance, especially in light of their high levels of first-line antibiotic resistance. These resistance patterns have important ramifications, particularly in areas with few empirical treatment alternatives and little utilization of laboratory-guided therapy.

***Specific Comments:***

***Introduction:***

***Line 12: The phrase "The bacterial isolates of B. cepacian” should be revised to “B. cepacian isolates” for conciseness and grammatical accuracy.***

Response to Reviewer:

Thank you for pointing out the phrasing issue in the Introduction. In the revised manuscript (Line 65, Page 2), we have updated the phrase from “The bacterial isolates of *B. cepacia*” to “*B. cepacia* isolates” for improved conciseness and grammatical accuracy.

***Page 4, Line 6: Please correct the phrase "by using the random sampling procedure..." by removing the extra period.***

Response to Reviewer:

Thank you for your careful observation. We have removed the extra period from the phrase *"by using the random sampling procedure..."* in the revised manuscript (Line 111, Page 4) to correct the punctuation.

***Page 4, Lines 8–20: The inclusion of general precautions for handling B. cepacia and A. sobria seems misplaced in the methodology section. The methods should focus on detailing the specific procedures you followed, not general laboratory guidelines.***

Response to Reviewer:

Thank you for your insightful comment. In response, we have revised the content in Lines 113–120 on Page 4 in the revised manuscript by removing the general biosafety precautions previously included for handling *B. cepacia* and *A. sobria*, as they were not directly related to the methodology of this study. The section now clearly describes the specific procedures followed, including the inclusion criteria for clinical specimens, types of samples collected (e.g., CSF, urine, blood, wound swabs), exclusion of contaminated samples, and the cultivation and incubation protocols used.

***Page 20, Lines 1–8: The current text—"In the end, these resistance profiles have significant clinical implications. They need the use of combination or second-line treatments, which could be more expensive, hazardous, or less effective."—should be revised for clarity and academic tone. Suggested revision:***

***"Ultimately, these resistance profiles carry significant clinical implications, often necessitating combination therapies or the use of second-line antibiotics, which may be more costly, associated with higher toxicity, or have reduced efficacy."***

Response to Reviewer:

Thank you for your suggestion to improve the clarity and tone of the statement. We have revised the sentence in Lines 485–488 on Page 20 in the revised manuscript as follows:

Ultimately, these resistance profiles carry significant clinical implications, often necessitating combination therapies or the use of second-line antibiotics, which may be more costly, associated with higher toxicity, or have reduced efficacy

***References: Several citations are outdated (e.g., references 3, 5, 17, 18, 25). It is advised to incorporate recent and relevant literature to strengthen the scientific foundation of the manuscript.***

Response to Reviewer:

We appreciate the reviewer’s observation regarding the use of outdated references. We have updated references 3, 5, 17, and 25 with more recent and relevant literature published within the last 5 years to better reflect current scientific knowledge. These updated references have been highlighted in the reference list for ease of review. However, reference 18 has been retained intentionally, as it provides the original primer sequences for *bla_GES_*, which are essential to the PCR methodology used in this study. No recent source offers an alternative validated primer set for this gene.

***While revising your submission, please upload your figure files to the Preflight Analysis and Conversion Engine (PACE) digital diagnostic tool, https://pacev2.apexcovantage.com/. PACE helps ensure that figures meet PLOS requirements. We upload all figures.***

Response to Editor:

We confirm that all figure files have been successfully uploaded to the [PACE (Preflight Analysis and Conversion Engine)](https://pacev2.apexcovantage.com/) digital diagnostic tool, as requested. The figures have been checked for compliance with PLOS formatting requirements, and any necessary adjustments have been made to meet the journal’s standards.

Thank you for providing this helpful tool to ensure figure quality and consistency.
